# Evaluation of ultrasonographic and clinicopathological features of patients with abnormal [^68^Ga]Ga-DOTA-TATE uptake in the thyroid gland

**DOI:** 10.1186/s13550-026-01406-y

**Published:** 2026-03-01

**Authors:** Beril Turan Erdogan, Fatma Dilek Dellal Kahramanca, Gulsum Karaahmetli, Seyda Turkolmez, Fazli Erdogan, Cevdet Aydin, Oya Topaloglu, Reyhan Ersoy, Bekir Cakir

**Affiliations:** 1 Department of Endocrinology and Metabolism, Ankara Bilkent City Hospital, Bilkent Avenue, Çankaya, Ankara, 06800 Turkey; 2 Department of Nuclear Medicine, Ankara Yildirim Beyazit University Faculty of Medicine, Ankara, Turkey; 3 Department of Pathology, Ankara Yildirim Beyazit University Faculty of Medicine, Ankara, Turkey; 4 Department of Endocrinology and Metabolism, Ankara Yildirim Beyazit University Faculty of Medicine, Ankara, Turkey

**Keywords:** [^68^Ga]Ga-DOTA-TATE, PET/CT, Thyroid uptake, Neuroendocrine tumors, Fine-needle aspiration biopsy

## Abstract

**Background:**

[^68^Ga]Ga-DOTA-TATE PET/CT is primarily used for imaging neuroendocrine tumors, yet incidental thyroid uptake is observed in 7–11% of scans. While focal uptake has been associated with an increased risk of malignancy, the ultrasonographic and pathological features of these cases remain underexplored. This study aimed to investigate the ultrasonographic and clinicopathological features of patients with abnormal [^68^Ga]Ga-DOTA-TATE uptake in the thyroid gland.

**Results:**

Among 2,971 patients who underwent [^68^Ga]Ga-DOTA-TATE PET/CT between April 2019 and November 2023, abnormal thyroid uptake was detected in 154 patients (5.2%). Of these, 39 patients with complete ultrasonographic evaluation constituted the final analytical cohort and were included in the detailed clinicopathological analysis. Uptake was focal in 59%, diffuse in 17.9%, and heterogeneous in 23.1% of cases. Fine-needle aspiration biopsy (FNAB) was performed in 24 patients, with 41.7% yielding non-diagnostic results. Only one case of papillary thyroid carcinoma was detected, corresponding to a 2.6% malignancy rate. Diffuse uptake was commonly linked to Hashimoto’s thyroiditis. A moderate inverse correlation was observed between SUVmax and BMI (*r* = − 0.54, *p* < 0.05), with a mean SUVmax of 7.35 ± 3.08.

**Conclusions:**

Focal [^68^Ga]Ga-DOTA-TATE thyroid uptake may suggest malignancy, though the observed malignancy rate in our cohort was lower than previously reported. Diffuse uptake was generally associated with benign inflammatory conditions. The inverse association between SUVmax and BMI is a novel finding warranting further investigation. Comprehensive evaluation, including ultrasonography and FNAB, is advised for patients with incidental focal uptake.

## Introduction

The thyroid gland plays a fundamental role in regulating various metabolic processes and is one of the most vascularized endocrine organs in the body. While incidental findings in the thyroid gland during imaging studies have become more frequent with the widespread use of advanced imaging technologies, such as positron emission tomography/computed tomography (PET/CT), the clinical significance of these findings remains a subject of ongoing research. [^68^Ga]Ga-DOTA-TATE PET/CT, initially developed for detecting and staging neuroendocrine tumors (NETs), is a highly sensitive imaging technique targeting somatostatin receptors (SSTRs), especially the subtype SSTR2, which is commonly expressed in NETs [1, 2]. However, incidental thyroid uptake of [^68^Ga]Ga-DOTA-TATE is increasingly observed, raising questions regarding its clinical interpretation and management [3, 4].

[^68^Ga]Ga-DOTA-TATE binds selectively to SSTRs, and this radiotracer has been extensively used to detect primary and metastatic NETs such as gastroenteropancreatic (GEP) NETs, pheochromocytoma, paraganglioma, and medullary thyroid carcinoma [5, 6]. While these tumors are the primary focus of [^68^Ga]Ga-DOTA-TATE PET/CT imaging, incidental findings of thyroid uptake in approximately 7–11% of scans present a diagnostic challenge [7, 8]. The thyroid gland, which may express SSTRs in both physiological and pathological conditions, demonstrates varying patterns of uptake, often detected as focal or diffuse accumulation during imaging [1, 9].

The potential causes of [^68^Ga]Ga-DOTA-TATE uptake in the thyroid gland range from benign conditions such as chronic thyroiditis and multinodular goiter to more serious pathologies, including papillary thyroid carcinoma and medullary thyroid carcinoma [10, 11]. Recent studies have demonstrated that approximately 20% of patients with focal [^68^Ga]Ga-DOTA-TATE uptake in the thyroid are diagnosed with malignancy, most commonly papillary or medullary thyroid carcinoma [1, 12]. Meanwhile, diffuse uptake is often associated with benign conditions like Hashimoto’s thyroiditis or multinodular goiter [6, 13]. Previous studies have explored the prevalence and diagnostic implications of incidental [^68^Ga]Ga-DOTA-TATE uptake [3, 12]. These findings suggest the need for a cautious and systematic approach to managing incidental thyroid uptake, particularly in cases of focal uptake where the risk of malignancy is higher [14, 15].

The presence of SSTRs in the thyroid, particularly in conditions involving chronic inflammation, may explain the frequent incidental findings in this gland [16]. This is particularly evident in autoimmune thyroid diseases like Hashimoto’s thyroiditis, where upregulated SSTR expression can lead to increased [^68^Ga]Ga-DOTA-TATE accumulation [6]. However, focal uptake remains a red flag for potential malignancy, underscoring the importance of a comprehensive diagnostic workup, including ultrasonography and fine-needle aspiration biopsy (FNAB), in these patients [13, 14].

## Materials and methods

**Fig. 1:**
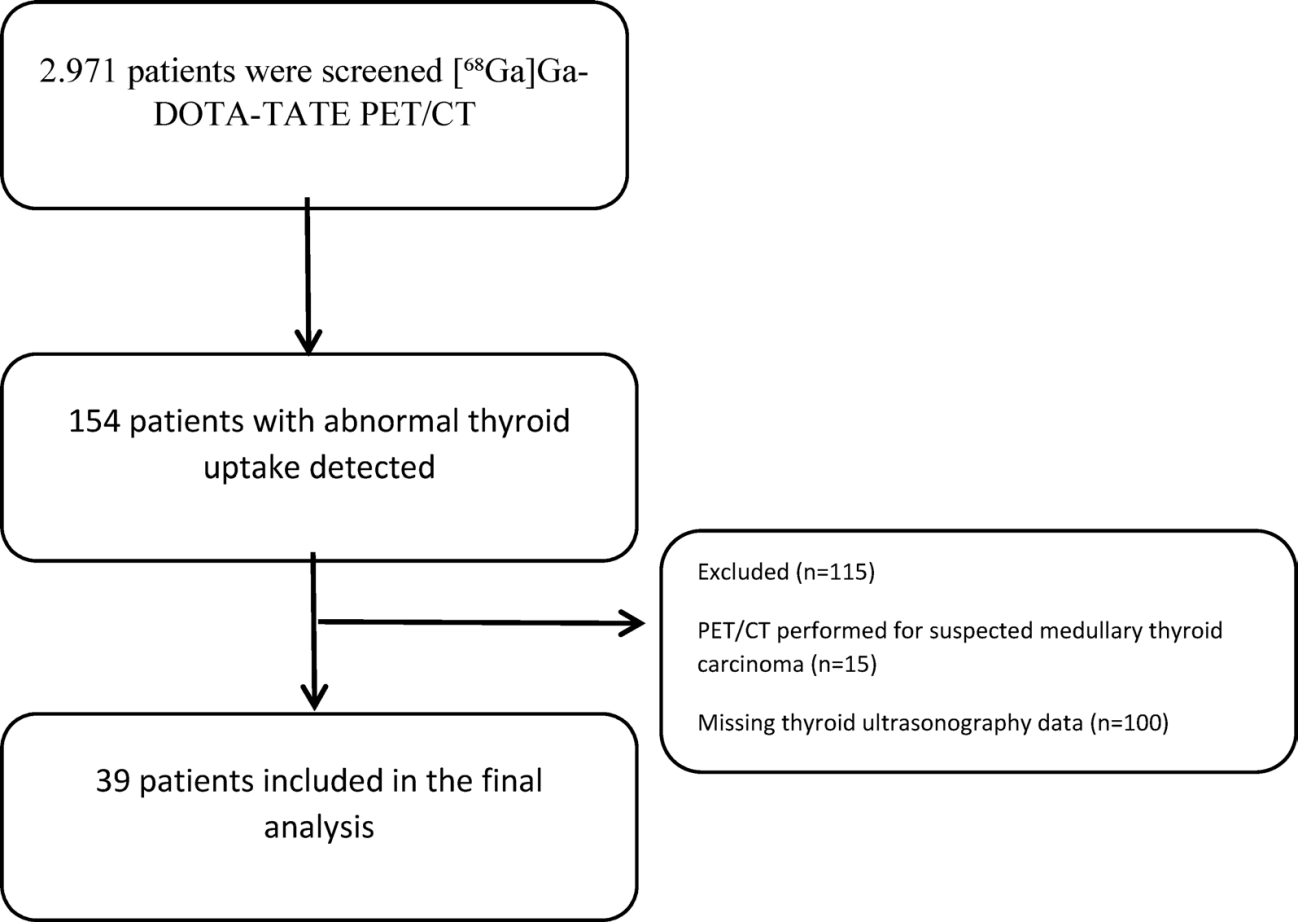
Flow diagram of patient selection

A total of 2971 patients who underwent [^68^Ga]Ga-DOTA-TATE PET/CT scans between April 2019 and November 2023 were screened in this retrospective study. Patients were evaluated based on the presence or absence of abnormal uptake in the thyroid gland. Only 154 of these patients exhibited [^68^Ga]Ga-DOTA-TATE uptake. Fifteen patients were excluded from the study because their PET-CT scans were performed with a preliminary diagnosis of medullary thyroid cancer (MTC), a condition for which [^68^Ga]Ga-DOTA-TATE PET/CT is a known and established imaging modality. Including these patients would have introduced selection bias by focusing on a group already known to demonstrate uptake. Additionally, 100 patients were excluded due to lack of available thyroid ultrasonography, which was essential for the study’s aim of evaluating ultrasonographic features and correlating them with PET findings. Ultimately, 39 patients with both [^68^Ga]Ga-DOTA-TATE uptake and complete thyroid ultrasonography data were included in the final analysis. The patient selection process from initial screening to the final analytical cohort is summarized in Fig. 1. Detailed thyroid disease history was obtained for all patients, including previous diagnoses of thyroiditis, nodular disease, or thyroid dysfunction. Treatment history, including thyroid hormone replacement, antithyroid medications, or previous interventions, was systematically recorded. During data collection, the following parameters potentially influencing [⁶⁸Ga]Ga-DOTA-TATE uptake were systematically recorded: current use of somatostatin analogues (SSAs), thyroid hormone replacement therapy (levothyroxine), antithyroid medications, recent exposure to iodinated contrast agents (within 4 weeks of PET imaging), and previous peptide receptor radionuclide therapy (PRRT). Treatment duration and dosing information were obtained from electronic medical records when available. Laboratory evaluation included serum thyroid stimulating hormone (TSH; normal range: 0.4-4.0 mIU/L), free T4 (fT4; normal range: 0.8–1.8 ng/dL), and free T3 (fT3; normal range: 2.3–4.2 pg/mL). Anti-thyroid peroxidase antibodies (Anti-TPO; normal < 60 U/mL) and anti-thyroglobulin antibodies (Anti-TG; normal < 4.5 IU/mL) were measured to assess autoimmune thyroid disease.

Patients with prior thyroidectomy or radioiodine therapy were excluded to eliminate potential confounding effects on [^68^Ga]Ga-DOTA-TATE uptake patterns. For patients with chronic thyroiditis, duration of disease and current treatment status were documented when available.Height and weight were routinely recorded in all patients undergoing PET/CT imaging as part of the standard clinical evaluation, and body mass index (BMI) was subsequently calculated. Given the inclusion of patients with paragangliomas or pheochromocytomas, hypertensive status was reviewed; however, all patients included in this analysis were normotensive at the time of imaging. The medical records of these 39 patients were reviewed retrospectively, and demographic, anthropometric, clinical, laboratory, and imaging findings were recorded. Notably, none of the included patients had a history of thyroidectomy or radioiodine treatment, thus reducing the potential impact of prior therapeutic interventions on [^68^Ga]Ga-DOTA-TATE uptake. The study was approved by the local ethics committee and conducted in accordance with the Helsinki Declaration.

[^68^Ga]Ga-DOTA-TATE PET/CT imaging was performed primarily in patients diagnosed with or suspected of having neuroendocrine tumors. An intravenous dose of [^68^Ga]Ga-DOTA-TATE was administered at a dose of 2 MBq (Megabecquerel) per kilogram of body weight, Whole-body PET/CT scans were performed approximately 60 min after injection. The images were evaluated by an experienced nuclear medicine specialist. Normal physiological uptake was not considered in this study; only abnormal uptake patterns (focal, diffuse, or heterogeneous) identified on PET/CT were evaluated. Uptake patterns were categorized using predefined criteria. Focal uptake was defined as tracer accumulation localized to a discrete area or nodule within the thyroid gland, with clear demarcation from surrounding thyroid tissue. Diffuse uptake was characterized by relatively uniform tracer distribution throughout both thyroid lobes, exceeding background activity. Heterogeneous uptake was defined as irregular or patchy tracer distribution affecting multiple distinct areas of the gland without uniform involvement. All PET/CT images were initially interpreted by an experienced nuclear medicine physician, and equivocal cases were reviewed by a second nuclear medicine specialist. In instances of discordant interpretation, consensus was reached through joint review. Uptake in the thyroid gland was assessed using the maximum standardized uptake value (SUVmax), and the degree of metabolic activity was compared among patients. Visual assessment was prioritized over predetermined SUVmax thresholds, as no universally accepted cutoff values exist for distinguishing physiological from pathological thyroid uptake. Uptake was considered abnormal when it appeared focal, asymmetric, or diffusely increased compared to background thyroid tissue, regardless of absolute SUVmax values.

The ultrasonographic evaluations were performed to detect the presence, size, and structure of thyroid nodules, as well as other morphological changes in the thyroid gland. During the ultrasound examination, the nodules were classified as cystic, hypoechoic, or isoechoic, and conditions such as multinodular goiter and chronic thyroiditis were identified.

FNAB was performed in nodules demonstrating suspicious ultrasonographic features (marked hypoechogenicity, irregular margins, microcalcifications, or increased vascularity) in combination with corresponding [⁶⁸Ga]Ga-DOTA-TATE uptake on PET/CT imaging. All procedures were performed by experienced endocrinologists using ultrasound guidance with a 25–27 gauge needle. For nodules yielding non-diagnostic cytology, repeat aspiration was typically performed within 4–6 weeks, allowing adequate time for cytopathological evaluation and patient rescheduling. The decision to perform repeat biopsy was individualized based on the degree of ultrasonographic suspicion, nodule size, patient clinical status, and the overall prognosis of the underlying primary malignancy. Nodules with persistently non-diagnostic results after repeated attempts were followed with serial ultrasonography at 6-month intervals when clinically appropriate. The decision for surgical intervention was determined by integrating cytological findings, ultrasonographic features, changes in nodule characteristics over time, patient adherence to follow-up, and overall clinical context. Importantly, given that many patients in this cohort had underlying neuroendocrine malignancies requiring ongoing systemic treatment, clinical management priorities were often necessarily weighted toward surveillance and treatment of the primary malignancy, which in some cases limited the feasibility of aggressive thyroid-specific interventions.

### Statistical analysis

Statistical analyses were performed using SPSS version 26.0 (IBM Corp., Armonk, NY, USA). Continuous variables were expressed as mean ± standard deviation (SD), while categorical variables were presented as frequencies and percentages. Variables with non-normal distribution were expressed as median (interquartile range). [^68^Ga]Ga-DOTA-TATE PET/CT findings were compared with ultrasonographic and clinical parameters. For group comparisons, the Student’s t-test was used for normally distributed data, while the Mann-Whitney U test was applied for non-normally distributed data. Chi-square or Fisher’s exact test was used to analyze categorical data. Spearman correlation analyses were conducted to evaluate relationships between variables. A p-value of less than 0.05 was considered statistically significant.

## Results

The study included a total of 39 patients, with a mean age of 56.8 years (± 15.52), of which 48.7% were male (*n* = 19). The patients had an average height of 167.1 cm (± 9.09) and an average weight of 82.3 kg (± 16.34), corresponding to a mean body mass index (BMI) of 29.53 kg/m² (± 5.71). The majority of the patients were either overweight or obese (Table 1). The mean systolic blood pressure was 124 mmHg (± 21) and the mean diastolic blood pressure was 65 mmHg (± 15).

**Table 1:** Demographic, Clinical and Biochemical Characteristics of Patients

	Patients *n* = 39
**Age (years), (SD)**	56.8 (15.52)
**Gender, male, n (%)**	19 (48.7)
**Height (cm), (SD)**	167.1 (9.09)
**Weight (cm), (SD)**	82.3 (16.34)
**BMI (kg/m** ^**2**^ **) (SD)**	29.53 (5.71)
**Systolic blood pressure (mmHg) (SD)**	124 (21)
**Diastolic blood pressure (mmHg) (SD)**	65 (15)
**TSH (mIU/L) (IQR)**	1.3(0.73–2.38)
**fT4 (pmol/L) (IQR)**	1.17(1-1.26)
**fT3 (pmol/L) (IQR)**	3.07 (2.83–3.55)
**Anti-TPO (IU/mL) (IQR)**	31 (28–48)
**Anti-TG (IU/mL) (IQR)**	1.3 (0.6–3.2)
**Thyroglobulin (ng/ml) (IQR)**	19.5 (3.7–56)
**[⁶⁸Ga]Ga-DOTA-TATE SUVmax**	7.35 ± 3.08
**Indication of [⁶⁸Ga]Ga-DOTA-TATE PET** Paraganglioma, n (%) Breast cancer, n (%) Pheochromocytoma, n (%) Lung neuroendocrine tumor, n (%) Insulinoma, n (%) Gastrointestinal neuroendocrine tumor, n (%) Bilaterally adrenal adenoma, n (%) Prostate cancer, n (%) Operated parathyroid adenoma, n (%) Rectal adenocarcinoma + renal cell carcinoma, n (%) Cholangiocelluler carcinoma, n (%)	1 (2.6) 1 (2.6) 8 (20.5) 2 (5.1) 1 (2.6) 13 (33.3) 1 (2.6) 9 (23.1) 1 (2.6) 1 (2.6) 1 (2.6)
**Department that requests [⁶⁸Ga]Ga-DOTA-TATE PET** Cardiology, n (%) Medical oncology, n (%) Endocrinology, n (%) Thoracic surgery, n (%) Gastroenterology, n (%) Nuclear medicine, n (%) Radiation oncology, n (%) Urology, n (%) Gastroenterology surgery, n (%)	1 (2.6) 9 (23.1) 11 (28.2) 1 (2.6) 4 (10.3) 3 (7.7) 3 (7.7) 6 (15.4) 1 (2.6)

Results are expressed as mean ± standard deviation, median (interquartile range) or frequency (%)

SD: standard deviation; BMI: body mass index; IQR: interquartile range; TSH: Thyroid-stimulating hormone; fT4: Free Thyroxine; fT3: Free Triiodothyronine; Anti-TPO: Anti-Thyroid Peroxidase Antibody; Anti-TG: Anti-Thyroglobulin Antibody; SUVmax: Maximum Standardized Uptake Value, PET: positron emission tomography

Regarding thyroid function, TSH median level was 1.3 mIU/L (0.73–2.38), fT4 median level was 1.17 pmol/L (1-1.26), and fT3 median level was 3.07 pmol/L (2.83–3.55). The thyroid autoantibody analysis revealed a significantly elevated Anti-TPO median level of 31 IU/mL (28–48), while Anti-TG median level was 1.3 IU/mL (0.6–3.2). The median thyroglobulin level was 19.5 ng/mL (3.7–56). (Table 1).

The indications for [^68^Ga]Ga-DOTA-TATE PET/CT scans included gastrointestinal NETs (33.3%), prostate cancer (23.1%) and pheochromocytoma (20.5%) as the most frequent reasons for imaging. Other indications included paraganglioma, breast cancer, lung neuroendocrine tumors, and, insulinoma each present in smaller proportions (Table 1). The majority of referrals for PET imaging came from the endocrinology department (28.2%), followed by medical oncology (23.1%) and urology (15.4%). SUVmax for [^68^Ga]Ga-DOTA-TATE PET/CT was 7.35 (± 3.08), indicating varying levels of metabolic activity in the thyroid tissue or nodule of these patients.

Among the 39 patients in the analytical cohort, thyroid hormone replacement with levothyroxine was documented in 7 patients (17.9%), all of whom had established diagnoses of primary hypothyroidism or chronic thyroiditis. Antithyroid medication (methimazole) was being used in 3 patients (7.7%) for management of hyperthyroidism. All patients receiving thyroid-specific medications (*n* = 10, 25.6%) demonstrated elevated thyroid autoantibodies. No patients were receiving somatostatin analogue therapy at the time of PET imaging. No patients had recent iodinated contrast exposure, and none had received prior PRRT.

[^68^Ga]Ga-DOTA-TATE uptake in the thyroid gland showed variable patterns, with 59% of patients exhibiting focal uptake, 17.9% showing diffuse uptake, and 23.1% displaying heterogeneous uptake patterns (Table 2). Focal uptake was the most common presentation. Anatomical distribution analysis showed that 30.8% of patients had uptake confined to the left lobe, while 28.2% had uptake in the right lobe. In 41.0% of cases, uptake could not be localized to a specific lobe due to diffuse, bilateral, or overlapping patterns.

**Table 2:** Characteristics of distribution of Ga uptake in Thyroid and Ultrasonographic Findings

	Patients *n* = 39
**Characteristics of distribution of Ga uptake**	
Focal, *n* (%)	23 (59.0)
Diffuse, *n* (%)	7 (17.9)
Heterogeneous, *n* (%)	9 (23.1)
**Involvement site** Right lobe, n (%) Left lobe, n (%) Diffuse/non-localizable, n (%)	11 (28.2) 12 (30.8) 16 (41)
**Ultrasonographic result** Nodular/multinodular goiter, n(%) --with chronic thyroiditis, n(%) -- without chronic thyroiditis, n(%) -Chronic thyroiditis, n(%)	35 (87.2) 8 (20.5) 27 (69.2) 4 (10.3)
**Ultrasonographic nodule feature** **Nodule structure, n** Solid, n (%), Mixed, n (%) **Echonegicity,** İsoechoic, n(%) Hypoechoic, n(%) Heterogeneous, n(%) **Presence of cystic degeneration**	*n* = 35 11 (31.4) 24 (68.6) 17 (48.6) 3 (8.6) 15(42.9) 24 (68.6)
**Nodule Sizes from FNAB** Nodule anteroposterior diameter (mm) (median, (IQR)) Nodule transvers diameter (mm) (median, (IQR)) Nodule longitudinal diameter (mm) (median, (IQR))	*n* = 45 13 (8.15–24.1) 16.6(12-25.55) 19.1 (14-31.35)

FNAB:Fine needle aspiration biopsy; IQR:Interquatile range

Ultrasonographic evaluation revealed that 35 patients (87.2%) had nodular or multinodular goiter. Among these, 20.5% were associated with chronic thyroiditis, whereas 69.2% had no signs of thyroiditis. Chronic thyroiditis without nodular involvement was identified in10.3% (Table 2). Regarding the ultrasonographic features of the nodules (*n* = 35), 31.4% were solid, and 68.6% exhibited a mixed structure. In terms of echogenicity, 48.6% of nodules were isoechoic, 8.6% were hypoechoic, and 42.9% had a heterogeneous echotexture. Additionally, cystic degeneration was present in 24 nodules (68.6%). The median dimensions of the nodules were as follows: anteroposterior diameter of 13 mm (8.15–24.1), transverse diameter of 16.6 mm (12–25.55), and longitudinal diameter of 19.1 mm (14–31.35). (Table 2).

FNAB was indicated in 30 (76.9%) of the patients, and it was performed in 24 (80%) of these cases. FNAB could not be performed in 3 patients due to the progression of their primary malignancies, and 3 patients declined the procedure. The median interval between PET/CT and ultrasound was 30 days (IQR: 13.5–72), between PET/CT and FNAB was 70 days (IQR: 19.75–124), and between ultrasound and FNAB was 30.5 days (IQR: 5.75–53.75).

Among the 45 nodules that underwent FNAB, the initial cytological distribution revealed 22 non-diagnostic results (48.9%), 18 benign (40.0%), 4 atypia of undetermined significance (8.9%), and 1 follicular neoplasm (2.2%). For the 22 nodules with initial non-diagnostic results, repeat FNAB was performed in 16 cases. Following repeat aspiration, 9 nodules yielded benign cytology, 3 remained non-diagnostic, and 4 were reclassified as atypia of undetermined significance (Table 3). Taking all biopsy attempts into account, the final cytological outcomes were: 13 nodules (28.9%) with persistently non-diagnostic results, 24 benign (53.3%), 7 atypia of undetermined significance (15.6%), and 1 follicular neoplasm (2.2%).

**Table 3:** Sequential Fine-Needle Aspiration Biopsy Results by Attempt Number

Location of the nodule	Right	20 (44.4)
	Left	25 (55.6)
**Nodule pathology result 1** (*n* = 45)	Non-diagnostic, n (%)	22 (48.9)
	Benign, n (%)	15 (33.3)
	Atypia of undetermined significance, n (%)	7 (15.6)
	Follicular neoplasm, n (%)	1 (2.2)
**Nodule pathology result 2** (*n* = 22)	Non-diagnostic, n (%)	7 (31.8)
	Benign, n (%)	7 (31.8)
	Atypia of undetermined significance, n (%)	8 (36.4)
**Nodule pathology result 3** (*n* = 8)	Non-diagnostic, n (%)	4 (50)
	Benign, n (%)	3 (37.5)
	Atypia of undetermined significance, n (%)	1 (12.5)
**Nodule pathology result 4** (*n* = 3)	Non-diagnostic, n (%)	1 (33.3)
	Benign, n (%)	0
	Atypia of undetermined significance, n (%)	2 (66.7)
**Nodule pathology result 5** (*n* = 1)	Non-diagnostic, n (%)	0
	Benign, n (%)	0
	Atypia of undetermined significance, n (%)	1 (100)

Sequential biopsies were performed when initial results were non-diagnostic or showed atypia requiring further evaluation

Of the 13 nodules with persistently non-diagnostic cytology after repeated attempts, 11 were followed with serial ultrasonography without significant interval growth or development of additional suspicious ultrasonographic features during available follow-up. Two patients were lost to thyroid-specific follow-up due to progression of their primary malignancies requiring transfer to palliative care. No additional surgical interventions were performed based on non-diagnostic cytology alone. One patient with repeatedly atypical cytology across three separate FNAB procedures underwent thyroidectomy, with final histopathology confirming papillary thyroid carcinoma; this represents the single confirmed malignancy in our cohort.

**Fig. 2:**
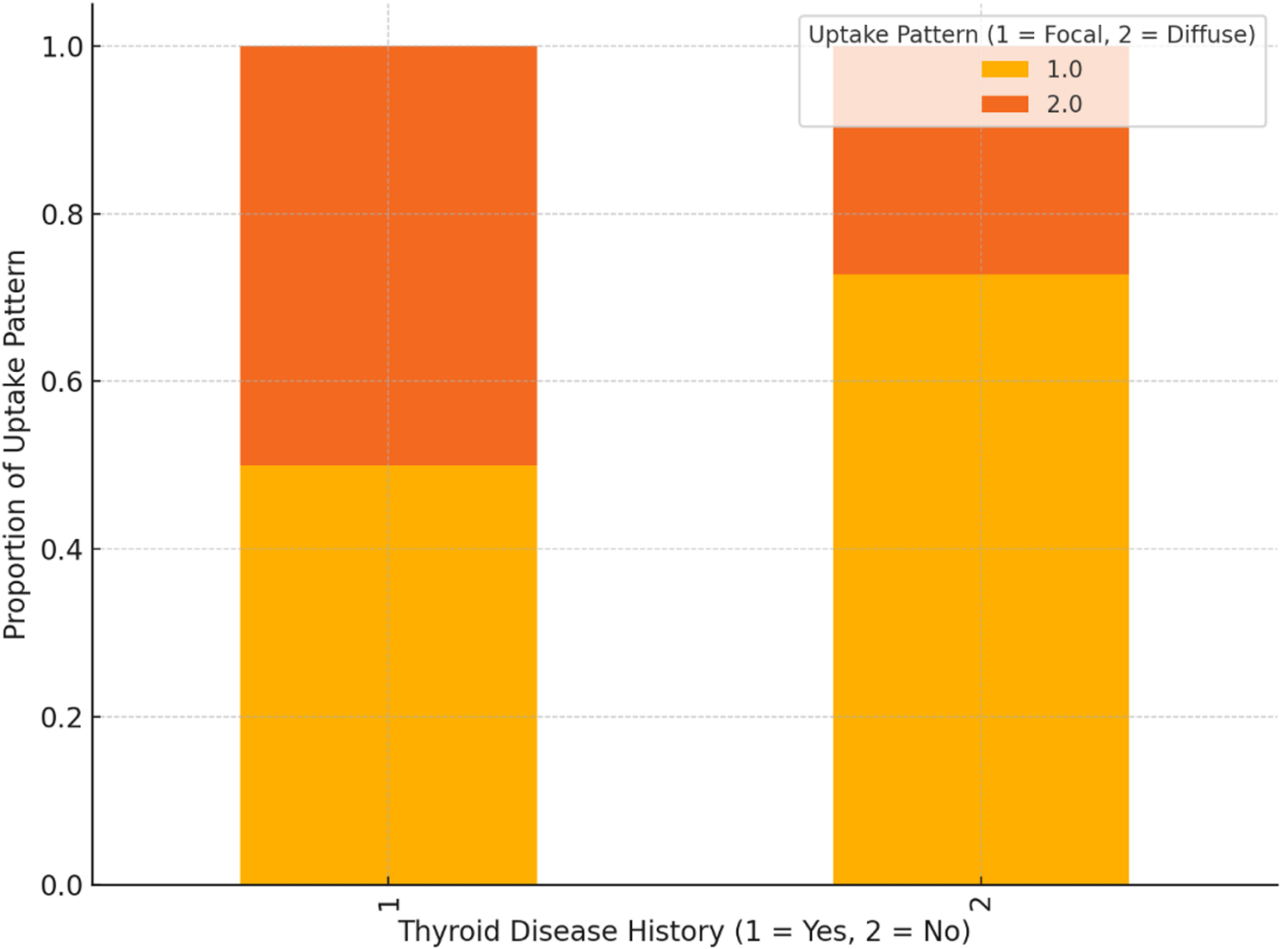
Distribution of Uptake Patterns Based on Thyroid Disease History

**Fig. 3:**
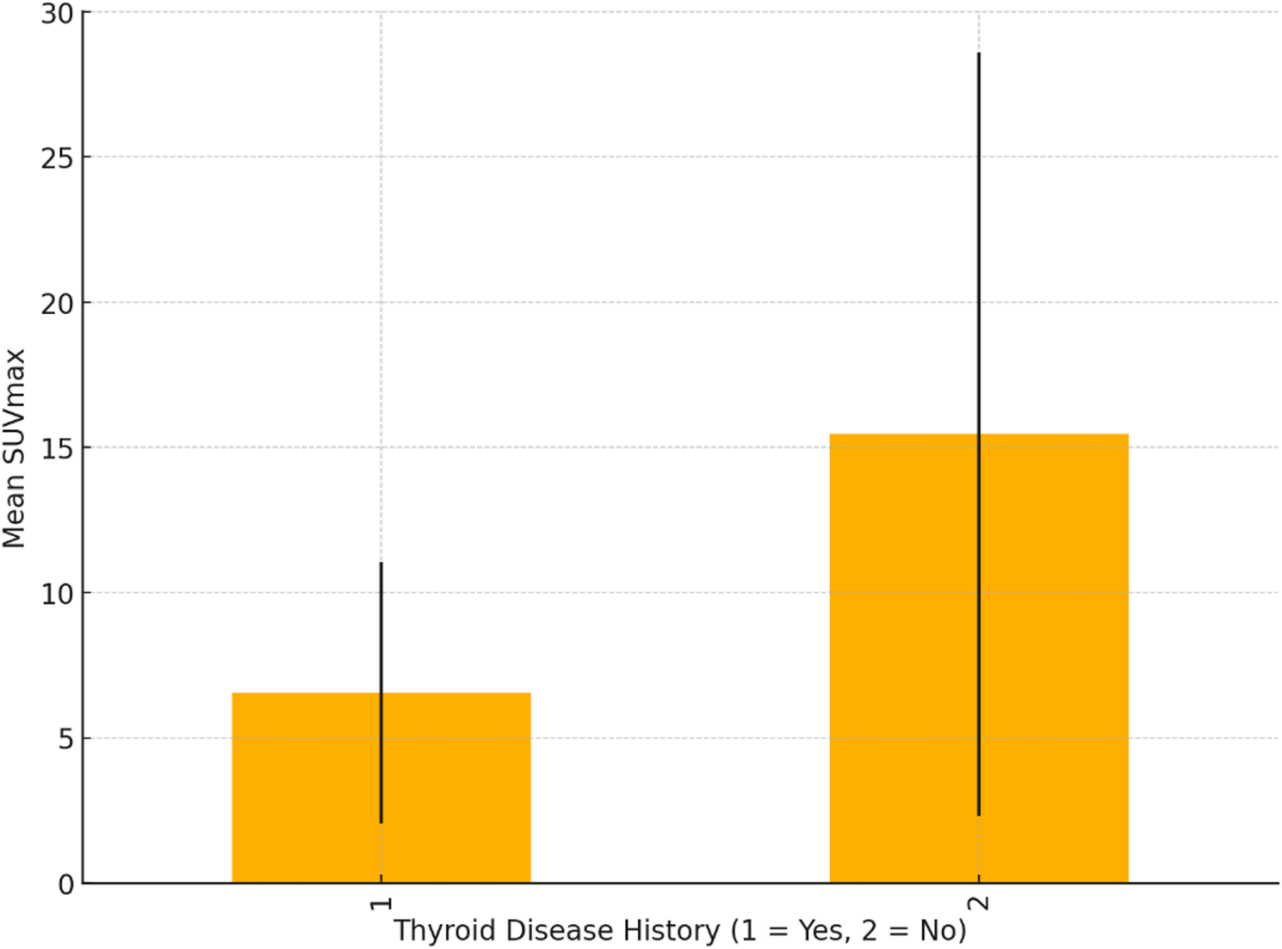
Mean SUVmax by Thyroid Disease History

A correlation analysis between SUVmax and patient characteristics revealed a weak to moderate negative correlation between SUVmax and both age (*r* = − 0.33) and BMI (*r* = − 0.54). In terms of thyroid disease history, patients with a known thyroid disease showed an equal distribution of focal and diffuse uptake (59%, 17.9% respectively), while patients without a known thyroid disease history exhibited predominantly focal uptake (72.7%). Furthermore, patients without thyroid disease had a higher mean SUVmax compared to those with a known thyroid disease, although this difference should be interpreted with caution due to the small sample size (SUVmax:8.07 vs. 6.94) (Figs. 2 and 3).

## Discussion

This study aimed to evaluate the clinicopathological and ultrasonographic features of patients with incidental [^68^Ga]Ga-DOTA-TATE uptake in the thyroid gland, an increasingly observed phenomenon in clinical practice. This study demonstrated that focal [^68^Ga]Ga-DOTA-TATE uptake in the thyroid gland is a potential indicator of malignancy, while diffuse uptake is predominantly associated with benign conditions, particularly autoimmune thyroid diseases such as Hashimoto’s thyroiditis.

Our findings both corroborate and extend previous observations regarding incidental thyroid uptake. While Nockel et al. reported malignancy rates of approximately 20% in focal uptake cases, our cohort demonstrated a lower rate (2.6%), which may reflect differences in patient populations, imaging protocols, or biopsy strategies. The predominance of focal uptake (59%) in our series aligns with earlier reports, though our comprehensive ultrasonographic correlation provides additional insight into the morphological characteristics of these lesions. Notably, our identification of an inverse relationship between SUVmax and BMI represents a novel finding that has not been systematically evaluated in previous thyroid uptake studies.

One of the key findings of this study is the predominance of focal [^68^Ga]Ga-DOTA-TATE uptake in the thyroid gland, observed in 59% of patients. This is a significant observation given that focal uptake is often associated with a risk of malignancy, as highlighted in several previous studies [1, 12]. The association between focal uptake and malignancy, particularly papillary and medullary thyroid carcinoma, underscores the importance of vigilant follow-up and comprehensive evaluation in patients with such findings. In our cohort, although only one case of papillary thyroid carcinoma was confirmed, the high percentage of focal uptake emphasizes the need for clinicians to remain cautious and consider further diagnostic procedures such as ultrasonography and FNAB. This aligns with previous literature suggesting that focal thyroid uptake should be treated as a potential indicator of malignancy [1, 13, 14, 15]. However, the absence of confirmed malignancy in our cohort also highlights the possibility of false positives or uptake related to benign but metabolically active thyroid processes. In clinical practice, it is essential to distinguish physiological background uptake of [^68^Ga]Ga-DOTA-TATE in the thyroid gland—typically low-grade and homogeneous—from abnormal patterns.

The interpretation of physiological uptake patterns in PET imaging varies depending on the specific radiotracer utilized. Studies employing different ^68^Ga-labeled peptides have demonstrated diverse background uptake profiles across various organs [16, 17], highlighting the importance of establishing tracer-specific reference ranges. While comprehensive normative data for thyroid uptake with somatostatin receptor-targeting agents remain limited, the physiological thyroid activity is generally characterized by low-grade, homogeneous patterns [18]. The mean SUVmax of 7.35 ± 3.08 in our cohort substantially exceeds these typical physiological values, supporting our interpretation that the detected uptake predominantly represents pathological rather than physiological activity.

Abnormal uptake, manifesting as focal, diffuse, or heterogeneous patterns, may indicate underlying pathological conditions. Our study specifically focused on such abnormal patterns that are more likely to be clinically significant. Recognizing this distinction is critical to avoid unnecessary interventions in cases of physiological uptake, while ensuring that potentially important abnormalities are not overlooked. Therefore, a cautious and individualized approach is necessary, balancing the potential risk with the need to avoid unnecessary procedures. Larger, prospective studies are required to define the true predictive value of focal uptake.

In contrast, diffuse uptake was observed in 17.9% of the patients and was predominantly linked to benign thyroid conditions such as Hashimoto’s thyroiditis. This finding is consistent with other studies that have shown diffuse [^68^Ga]Ga-DOTA-TATE uptake to be more commonly associated with benign inflammatory conditions [6, 13]. The diffuse uptake likely reflects the increased expression of SSTRs in inflamed thyroid tissues, a phenomenon that is particularly evident in autoimmune thyroid conditions such as Hashimoto’s thyroiditis [18]. The upregulation of SSTRs in chronic inflammation may explain the heightened radiotracer accumulation in these cases. These findings support the hypothesis that diffuse uptake, while noteworthy, is more likely to indicate benign pathology, thereby distinguishing it from the more concerning focal uptake patterns.

Another significant finding of our study is the inverse correlation between BMI and SUVmax in thyroid imaging using [⁶⁸Ga]Ga-DOTA-TATE (*r* = − 0.54, *p* < 0.05). This preliminary observation requires cautious interpretation given the modest sample size and heterogeneous cohort, but suggests a potential relationship between body composition and SSTR expression influenced by metabolic and inflammatory factors.

In our cohort, no patients were receiving somatostatin analogue therapy, had undergone peptide receptor radionuclide therapy (PRRT), or had recent iodinated contrast exposure, excluding these as major confounders. Thyroid-specific medications were used in 25.6% of patients, all with elevated autoantibodies, and chronic thyroiditis was present in 30.8%, indicating an inflammatory background that may affect SSTR2 expression.

[⁶⁸Ga]Ga-DOTA-TATE targets somatostatin receptors, particularly SSTR2, which are modulated under metabolic and inflammatory conditions. Obesity is associated with low-grade systemic inflammation and altered adipokine profiles, which may influence receptor expression [19, 20]. The higher prevalence of subclinical hypothyroidism and nodular thyroid disease in individuals with elevated BMI may further contribute to this association [21, 22]. Technical factors related to image attenuation in obese patients may also partially affect SUV measurements despite attenuation correction.

Given its exploratory nature, the SUVmax–BMI correlation should be regarded as hypothesis-generating. Its clinical significance remains uncertain and warrants confirmation in larger cohorts with multivariate analyses to determine whether BMI independently influences thyroid SSTR expression or reflects confounding by metabolic and inflammatory factors. A weak inverse correlation between SUVmax and age was also observed, suggesting possible age-related changes in thyroid metabolic activity [4].

FNAB was indicated in 76.9% of patients based on suspicious ultrasonographic features combined with [⁶⁸Ga]Ga-DOTA-TATE uptake. The initial non-diagnostic rate of 48.9% decreased to 28.9% after repeat procedures, demonstrating the value of sequential sampling. While this final rate remains modestly above the 20–30% benchmark from high-volume thyroid centers, several patient-specific factors likely contributed [23].

Our cohort consisted primarily of patients with active or previously treated neuroendocrine malignancies, many receiving systemic therapies that may affect tissue quality and sampling success. The presence of concurrent chronic thyroiditis in 20.5% of cases likely increased technical difficulty due to background fibrosis and hypocellularity factors demonstrated to increase non-diagnostic rates in Hashimoto’s thyroiditis [24]. Additionally, management priorities in this population were necessarily weighted toward the primary malignancy, influencing the intensity of thyroid-specific follow-up.

Importantly, among first biopsied nodules yielding diagnostic results, 37.5% were benign, and none of the nodules with persistently non-diagnostic cytology demonstrated concerning progression during follow-up. Surgical intervention was reserved for the single case with consistently atypical features across multiple biopsies that proved to be papillary thyroid carcinoma. This outcome suggests that while our non-diagnostic rate reflects real-world constraints in this complex oncologic population, clinically significant thyroid malignancies were appropriately identified through systematic repeat sampling and ultrasonographic surveillance.

The distribution of uptake patterns based on thyroid disease history also provides valuable clinical insights. In patients without a documented thyroid disease history, focal uptake was more common (72.7%), whereas those with known thyroid disease exhibited nearly equal distribution of focal and diffuse uptake. Furthermore, patients without pre-existing thyroid conditions demonstrated higher mean SUVmax values compared to those with a history of thyroid disease, suggesting that incidental focal uptake in otherwise asymptomatic individuals warrants careful investigation. This observation aligns with the current understanding that SSTR expression in the thyroid may vary depending on the underlying pathological state, and incidental findings in patients without a known thyroid disorder could be of greater concern.

An important observation from our study is the extremely low overall malignancy yield of incidental [⁶⁸Ga]Ga-DOTA-TATE thyroid uptake. Among 2,971 patients screened, only one thyroid malignancy was identified, corresponding to an overall prevalence of 0.034%. This rate is substantially lower than commonly reported malignancy rates for thyroid incidentalomas detected on FDG-PET imaging, which typically range from 14% to 35% in various cohorts. The marked difference likely reflects fundamental distinctions in tracer mechanisms: FDG accumulates in metabolically active malignant cells, whereas [⁶⁸Ga]Ga-DOTA-TATE targets somatostatin receptors that are frequently upregulated in benign inflammatory conditions, particularly autoimmune thyroiditis. This biological difference has important clinical implications. While FDG-avid thyroid incidentalomas warrant aggressive evaluation due to their higher malignancy risk, our findings suggest that [⁶⁸Ga]Ga-DOTA-TATE uptake—particularly when diffuse—more commonly reflects benign pathology. Therefore, the clinical approach to incidental thyroid findings on [⁶⁸Ga]Ga-DOTA-TATE PET/CT should be individualized, carefully weighing the benefits of further diagnostic workup against patient prognosis, underlying disease burden, and life expectancy. In patients with advanced neuroendocrine malignancies undergoing palliative treatment, aggressive pursuit of incidental thyroid findings may not be appropriate. Conversely, in younger patients with favorable prognoses, systematic evaluation including ultrasonography and selective FNAB remains justified, particularly for focal uptake patterns.

Several limitations merit acknowledgment in interpreting our findings. The retrospective design inherently restricts our ability to standardize follow-up protocols and may introduce selection effects, particularly given that our cohort represents patients who underwent both PET imaging and subsequent thyroid evaluation. Our sample size of 39 patients, while reflecting the relative rarity of this clinical scenario, limits statistical power for subgroup analyses and definitive conclusions regarding malignancy risk. The substantial rate of non-diagnostic FNAB results further constrains pathological confirmation of our imaging observations.

Furthermore, our patient population was heterogeneous, encompassing various primary conditions that led to [^68^Ga]Ga-DOTA-TATE imaging, which may have influenced thyroid uptake patterns independently. The absence of systematic comparison with conventional thyroid imaging modalities or standardized long-term follow-up prevents comprehensive assessment of the clinical trajectory of these incidental findings. Additionally, variations in PET imaging protocols and reconstruction parameters across the study period may have affected SUVmax measurements, though all scans were performed on the same equipment with consistent acquisition protocols. In addition, SUVmax measurements were not normalized to a reference tissue such as liver or blood pool activity, which may limit inter-individual comparability and could have influenced the observed correlation with BMI.

The limited sample size in our study reflects both the relatively low incidence of incidental thyroid uptake and the requirement for comprehensive thyroid evaluation in our analysis. While this constraint affects the statistical power of our observations, particularly for rare outcomes such as malignancy, it provides a realistic representation of what clinicians encounter in routine practice. The single malignancy case identified emphasizes the importance of systematic evaluation while highlighting that most incidental findings represent benign conditions.

## Conclusion

Our study provides several clinically relevant observations regarding [^68^Ga]Ga-DOTA-TATE uptake in the thyroid gland. First, focal uptake—while often associated with a higher risk of malignancy—did not correspond to any confirmed carcinoma cases in our cohort, highlighting the importance of cautious interpretation and further diagnostic assessment. Second, diffuse uptake was more frequently observed in patients with benign conditions, particularly autoimmune thyroid diseases such as Hashimoto’s thyroiditis, supporting its likely non-malignant nature. Third, the inverse relationship between SUVmax and BMI suggests a potential metabolic influence on somatostatin receptor expression, though this association requires validation in larger, prospective cohorts. As advanced imaging modalities become increasingly integrated into routine clinical practice, a deeper understanding of incidental findings will be essential for guiding appropriate diagnostic strategies and optimizing patient management.

## Data Availability

The datasets generated and/or analyzed during the current study are not publicly available due to privacy and ethical concerns but are available from the corresponding author on reasonable request.

## Abbreviations

BMI: Body Mass Index
FNAB: Fine Needle Aspiration Biopsy
fT3: Free Triiodothyronine
fT4: Free Thyroxine
NET: Neuroendocrine Tumor
PET/CT: Positron Emission Tomography / Computed Tomography
SD: Standard Deviation
SSTR: Somatostatin Receptor
SUVmax: Maximum Standardized Uptake Value
TSH: Thyroid-Stimulating Hormone
Anti-TPO: Anti-Thyroid Peroxidase Antibody
Anti-TG: Anti-Thyroglobulin Antibody
PRRT: Peptide receptor radionuclide therapy
SSA: Somatostatin analogue
